# Selection of an appropriate empiric antibiotic regimen in hematogenous vertebral osteomyelitis

**DOI:** 10.1371/journal.pone.0211888

**Published:** 2019-02-08

**Authors:** Ki-Ho Park, Dong Youn Kim, Yu-Mi Lee, Mi Suk Lee, Kyung-Chung Kang, Jung-Hee Lee, Seong Yeon Park, Chisook Moon, Yong Pil Chong, Sung-Han Kim, Sang-Oh Lee, Sang-Ho Choi, Yang Soo Kim, Jun Hee Woo, Byung-Han Ryu, In-Gyu Bae, Oh-Hyun Cho

**Affiliations:** 1 Division of Infectious Diseases, Department of Internal Medicine, Kyung Hee University Hospital, Kyung Hee University School of Medicine, Seoul, Republic of Korea; 2 Department of Orthopedic Surgery, Kyung Hee University Hospital, Kyung Hee University School of Medicine, Seoul, Republic of Korea; 3 Division of Infectious Diseases, Department of Internal Medicine, Dongguk University Ilsan Hospital, University of Dongguk College of Medicine, Goyang-si, Republic of Korea; 4 Department of Infectious Diseases, Busan Paik Hospital, Inje University College of Medicine, Busan, Republic of Korea; 5 Department of Infectious Diseases, Asan Medical Center, University of Ulsan College of Medicine, Seoul, Republic of Korea; 6 Department of Internal Medicine, Gyeongsang National University Hospital, Gyeongsang National University College of Medicine, Jinju, Republic of Korea; 7 Department of Internal Medicine, Gyeongsang National University Changwon Hospital, Changwon, Republic of Korea; 8 Institute of Health Sciences, Gyeongsang National University College of Medicine, Jinju, Republic of Korea; Eugene Lang College of Liberal Arts at the New School, UNITED STATES

## Abstract

**Background:**

Empiric antibiotic therapy for suspected hematogenous vertebral osteomyelitis (HVO) should be initiated immediately in seriously ill patients and may be required in those with negative microbiological results. The aim of this study was to inform the appropriate selection of empiric antibiotic regimens for the treatment of suspected HVO by analyzing antimicrobial susceptibility of isolated bacteria from microbiologically proven HVO.

**Method:**

We conducted a retrospective chart review of adult patients with microbiologically proven HVO in five tertiary-care hospitals over a 7-year period. The appropriateness of empiric antibiotic regimens was assessed based on the antibiotic susceptibility profiles of isolated bacteria.

**Results:**

In total, 358 cases of microbiologically proven HVO were identified. The main causative pathogens identified were methicillin-susceptible *Staphylococcus aureus* (33.5%), followed by methicillin-resistant *S*. *aureus* (MRSA) (24.9%), *Enterobacteriaceae* (19.3%), and *Streptococcus* species (11.7%). Extended spectrum β-lactamase (ESBL)-producing *Enterobacteriaceae* and anaerobes accounted for only 1.7% and 1.4%, respectively, of the causative pathogens. Overall, 73.5% of isolated pathogens were susceptible to levofloxacin plus rifampicin, 71.2% to levofloxacin plus clindamycin, and 64.5% to amoxicillin-clavulanate plus ciprofloxacin. The susceptibility to these oral combinations was lower in cases of healthcare-associated HVO (52.6%, 49.6%, and 37.6%, respectively) than in cases of community-acquired HVO (85.8%, 84.0%, and 80.4%, respectively). Vancomycin combined with ciprofloxacin, ceftriaxone, ceftazidime, or cefepime was similarly appropriate (susceptibility rates of 93.0%, 94.1%, 95.8%, and 95.8%, respectively).

**Conclusions:**

Based on our susceptibility data, vancomycin combined with a broad-spectrum cephalosporin or fluoroquinolone may be appropriate for empiric treatment of HVO. Fluoroquinolone-based oral combinations may be not appropriate due to frequent resistance to these agents, especially in cases of healthcare-associated HVO.

## Introduction

The incidence of vertebral osteomyelitis has increased over recent years, likely due to longer life expectancies, higher prevalence of chronic disease, and more effective diagnostic techniques [1–4]. Furthermore, healthcare-associated infections, such as catheter-related and device-related bloodstream infections, also increase the risk of vertebral osteomyelitis [5,6]. Empiric antibiotic therapy should be withheld until a microbiological diagnosis is confirmed, but it should be provided immediately to septic patients or those with neurologic compromise [7,8]. In addition, patients with no microbiological diagnosis after appropriate diagnostic tests may require empiric antibiotic treatment. Some authors recommended a second percutaneous needle biopsy in these patients [8,9]. An open biopsy may provide a higher microbiological yield than percutaneous needle biopsy [10–12]. Despite these efforts, the causative microorganisms are not identified in as many as 17–50% of cases [2,4,12–15]. Although the reason for this is uncertain, biopsy culture yield may be reduced by prior antibiotic use [13,16], non-purulent samples (i.e., bone tissue compared with soft tissue abscess) [17], and small volume of aspiration samples [18].

The selection of an empiric antimicrobial regimen for vertebral osteomyelitis should be based on consideration of the most likely causative pathogens and knowledge of local susceptibility patterns. Fluoroquinolone-based oral combinations were frequently used or recommended for empiric treatment for culture-negative vertebral osteomyelitis [1,2,19,20]. However, these studies were conducted in an epidemiologic setting featuring a low prevalence of methicillin-resistant *Staphylococcus aureus* (MRSA) [2,19,20], and the appropriateness of these regimens in high-MRSA-prevalence settings was not evaluated. The aim of this study was to evaluate the appropriateness of empiric antibiotic regimens suggested for treatment of hematogenous vertebral osteomyelitis (HVO) using the susceptibility data of pathogens isolated from cases of culture-proven HVO. We hypothesized that patients with healthcare-associated HVO would more frequently be infected with antibiotic-resistant organisms than those with community-acquired HVO. Therefore, the appropriateness of empiric antibiotic regimens was also assessed according to the risk of healthcare-associated infection.

## Methods

### Study design and setting

This observational cohort study was undertaken at five tertiary-care hospitals in the Republic of Korea, and included all adult patients diagnosed with HVO from January 2005 to December 2012. The study was conducted using the format recommended by the Strengthening the Reporting of Observational Studies in Epidemiology (STROBE) guidelines [21]. This study was approved by the Institutional Review Board of Gyeongsang National University Changwon Hospital (GNUCH 2018-05-010). Informed consent was waived because of the retrospective nature of the study.

### Inclusion and exclusion criteria

Adult patients (≥18 years of age) who presented with microbiologically proven HVO were included in this study. The diagnosis of HVO was established using previously published criteria [22,23], which included compatible clinical features, radiological evidence of vertebral osteomyelitis, and microbiologic demonstration of bacterial pathogens, either from the site of infection itself (*e*.*g*., abscess, intervertebral disc, or vertebral bone) or in the blood. Possible skin contaminants, such as coagulase-negative staphylococci (CoNS) and *Propionibacterium acnes*, were considered as true pathogens if they were isolated from ≥2 surgical, percutaneous biopsy, or blood cultures. Cases were excluded if there was a nonhematogenous source of vertebral infection, which included (1) penetrating trauma, (2) previously placed hardware, (3) laminectomy within 1 year prior to the vertebral osteomyelitis diagnosis, or (4) the presence of a stage 3–4 decubitus ulcer at the time of diagnosis [22,23]. Cases of tuberculous, brucellar, and fungal vertebral osteomyelitis were excluded using mycobacterial culture, nucleic acid amplification test for *Mycobacterium tuberculosis*, histopathologic examination, *Brucella* serology, and fungal culture.

### Data collection

Medical records were reviewed retrospectively for demographic information, underlying illness/conditions, presumed source of infection, diagnostic work-up, clinical presentation, and laboratory and radiological data. The present study builds on our previous work on the optimal duration of antibiotic therapy in patients with HVO [23].

### Microbiological analysis

The species and susceptibility profiles of all isolates were confirmed using either the Vitek2 (bioMérieux, Marcy-l'Etoile, France) or Microscan (Dade Behring Inc., Deerfield, IL) automated systems. The susceptibility of isolates pathogens to 15 antibiotics was assessed (amoxicillin, amoxicillin-clavulanate, ciprofloxacin, levofloxacin, clindamycin, rifampin, trimethoprim-sulfamethoxazole, fusidic acid, cefazolin, ceftriaxone, ceftazidime, cefepime, vancomycin, teicoplanin, and linezolid). Isolated strains were categorized by the Clinical Laboratory Standard Institute (CLSI) guidelines as susceptible, intermediate, or resistant to antimicrobial agents[24,25]. Prior to 2010, susceptibility breakpoints for cefazolin, ceftriaxone, and ceftazidime were ≤8 mg/L for *Enterobacteriaceae* [24]. In 2010, CLSI reduced breakpoints to ≤2 mg/L for cefazolin, ≤1 mg/L for ceftriaxone, and ≤4 mg/L for ceftazidime [25]. Cefepime susceptibility breakpoint for *Enterobacteriaceae* was 8 mg/L during entire study period [25]. Given the risk of treatment failure for intermediate strains, strains classified “intermediate” were reclassified as resistant. If the susceptibility results for surgery, percutaneous biopsy, and blood cultures were different, overall susceptibility was classified resistant.

We evaluated the overall susceptibility of eight antibiotics, alone or in combination, for empiric treatment of HVO, which were used or recommended in previous literatures. They included three fluoroquinolone-based oral combinations (levofloxacin plus rifampin, levofloxacin plus clindamycin, ciprofloxacin plus amoxicillin-clavulanate) [1,2,19,20], four vancomycin-based intravenous combinations (vancomycin plus ciprofloxacin, ceftriaxone, ceftazidime, and cefepime) [8,26], and cefazolin monotherapy [14,27]. For *Staphylococcus* species, overall susceptibility to fluoroquinolone-based combinations was classified as susceptible if the organisms were susceptible to both antimicrobial agents (except for clindamycin, which can be used as a monotherapy on *Staphylococcus* species) [8]. For all other species, strains were considered susceptible if they showed susceptibility to at least one of the antimicrobial agent combinations.

### Definitions

HVO was classified as community-acquired or healthcare-associated HVO according to published criteria [5]. Healthcare-associated HVO was defined as (i) onset of symptoms after 1 month of hospitalization with no evidence of vertebral osteomyelitis at admission, (ii) hospital admission within 6 months before symptom onset, or (iii) ambulatory diagnostic or therapeutic manipulations within 6 months before symptom onset (long-term central venous catheter use, arteriovenous fistula for hemodialysis, invasive intravascular techniques, urological, gynecological or digestive procedures, and cutaneous manipulations). Cases that did not meet any of the above criteria were classified as community-acquired HVO [5].

### Statistical analyses

Categorical variables are expressed as numbers and percentages and were compared by χ2 or Fisher’s exact test. Continuous variables are expressed as medians and interquartile ranges and were compared using Mann-Whitney *U* test. All statistical tests were two-tailed, and *P* ≤ 0.05 was considered to indicate statistical significance. All statistical analyses were performed using SPSS for Windows software (ver. 18.0; SPSS, Inc., Chicago, IL).

## Results

In total, 370 patients with microbiologically proven HVO were identified during the study period. Of the 370 cases, 12 were excluded due to incomplete medical records and 358 were included in the final analysis.

### Patients’ characteristics

The demographic and baseline characteristics of the patients are displayed in Table 1. The median age of the cohort was 65 years, and 186 (52.0%) were males. The most common underlying condition was diabetes (29.3%), followed by liver cirrhosis (9.2%) and malignancy (8.7%). According to the predefined criteria, 225 (62.8%) cases were community-acquired HVO and 133 (37.2%) were healthcare-associated HVO.

**Table 1:** Demographic and baseline characteristics of 358 patients with hematogenous vertebral osteomyelitis.

Variable	All patients (*n* = 358)
Age, median years (IQR)	65 (58–72)
Male sex	186 (52.0)
Underlying illness/conditions	
Diabetes mellitus	105 (29.3)
Liver cirrhosis	33 (9.2)
Malignancy	31 (8.7)
Immunosuppression	20 (5.6)
End-stage renal disease	15 (4.2)
Rheumatic disease	12 (3.4)
Healthcare-associated HVO	133 (37.2)
Presumed source of infection	
Urinary tract	35 (9.7)
Skin and subcutaneous tissues	30 (8.4)
Intraabdominal	26 (7.3)
Infected vascular access	25 (7.0)
Endocarditis	19 (5.3)
Unknown	223 (62.3)
Clinical data	
Time to diagnosis, median days (IQR)	22 (8–40)
Back pain	319 (89.1)
Body temperature > 38°C	190 (53.1)
Neurologic deficit at diagnosis	61 (17.0)
Concurrent metastatic infection	46 (12.8)
Laboratory data	
White blood cell count, ×10^9/^L, median (IQR)	114 (79–162)
C-reactive protein, mg/dL, median (IQR)	13 (6–22)
Erythrocyte sedimentation rate, mm/h, median (IQR)^a^	76 (55–100)
Positive blood cultures	265/339 (78.2)
Radiologic data	
Involvement of > 2 vertebral bodies	125 (34.9)
Involvement of cervical spine	31 (8.7)
Involvement of thoracic spine	85 (23.7)
Involvement of lumbosacral spine	283 (79.1)
Epidural involvement^b^	194 (54.2)
Paravertebral involvement^b^	192 (53.6)
Psoas involvement^b^	126 (35.2)

Data are no. (%) of patients, unless otherwise indicated.

Abbreviations: HVO, hematogenous vertebral osteomyelitis; IQR, interquartile range

^a^ Measured in 287 patients.

^b^ Either abscess or phlegmon.

### Microbiological findings

Of the 358 cases with microbiologically proven HVO, 93 (26.0%) were identified by diagnostic biopsy, 173 (48.3%) by blood cultures, and 92 (25.7%) by both. The most frequently isolated organisms were methicillin-susceptible *Staphylococcus aureus* (MSSA) (33.5%), follow by MRSA [24.9%]), *Enterobacteriaceae* (19.3%), and *Streptococcus* species (11.7%; Table 2). Of 42 *Streptococcus* species, viridans group streptococci were the most frequently isolated organisms (*n* = 21), followed by *Streptococcus agalactiae* (*n* = 15), *S*. *pneumoniae* (*n* = 4), and other streptococci (*n* = 2). CoNS, *Enterococcus* species, anaerobes, *Pseudomonas* species, and polymicrobial organisms accounted for 2.8%, 2.8%, 1.4%, 1.4%, and 0.8%, respectively.

**Table 2:** Antibiotic susceptibility testing results for 358 isolated microorganisms.

Organism	Percentage of isolates susceptible to														
	AMX	AMX -CL	CIP	LEVO	CLM	RIF	TMP-SMX	FA^a^	CFZ	CTR	CAZ	FEP	VAN	TEC	LZD
Methicillin-susceptible *S*. *aureus* (*n* = 120)	14.3	0	94.2	95.8	88.3	97.5	100	74.1	100	NA	NA	NA	100	100	100
Methicillin-resistant *S*. *aureus* (*n* = 89)	0	100	53.9	56.2	41.6	77.5	92.1	81.2	0	NA	NA	NA	100	100	100
Coagulase-negative staphylococci (*n* = 10)	30.0	50.0	70.0	70.0	70.0	80.0	80.0	NA	50.0	NA	NA	NA	100	100	100
*Streptococcus* species (*n* = 42)	90.5	95.2	NA	90.5	76.2	NA	NA	NA	100b	95.2	NA	NA	100	100	100
*Enterococcus* species (*n* = 10)	80.0	90.0	NA	NA	NA	NA	NA	NA	0	NA	NA	NA	100	100	100
*Enterobacteriaceae* (*n* = 69)	27.5	76.8	75.4	75.4	NA	NA	84.1	NA	82.6	91.3	91.3	91.3	NA	NA	NA
*Pseudomonas aeruginosa* (*n* = 5)	NA	0	100	100	NA	NA	0	NA	0	0	100	100	NA	NA	NA
Other (*n* = 13)^c^	NA	46.2	30.8	30.8	NA	NA	NA	NA	0	15.4	23.1	23.1	NA	NA	NA

Abbreviations: AMX, amoxicillin; AMX-CL, amoxicillin-clavulanate; CFP, cefepime; CAZ, ceftazidime; CFZ, cefazolin; CIP, ciprofloxacin; CLM, clindamycin; CTR, ceftriaxone; FA, fusidic acid; LEVO, levofloxacin; LZD, linezolid; MRSA, methicillin-resistant *S*. *aureus*; MSSA, methicillin-susceptible *S*. *aureus*; RIF, rifampin; TEC, teicoplanin; TMP-SMX; trimethoprim-sulfamethoxazole; VANC, vancomycin.

^a^ Susceptibility data for fusidic acid against methicillin-susceptible *S*. *aureus* and methicillin-resistant *S*. *aureus* isolates were available in 112 and 85 cases, respectively.

^b^ Excluding 25 streptococcal isolates without susceptibility data (21 viridans group streptococci and 4 *S*. *pneumoniae*); the remaining 17 *Streptococcus* isolates were susceptible to cefazolin.

^c^ Included *Bacteroides fragilis* (*n* = 2), *Bacteroides ureolyticus* (*n* = 1), *Prevotella melaninogenica* (*n* = 1), *Prevotella oralis* (*n* = 1), *Micrococcus* species (*n* = 1), *Pseudomonas putida* (*n* = 1), *Burkholderia cepacia* (*n* = 1), *Aggregatibacter aphrophilus* (*n* = 1), *Chryseobacterium meningosepticum* (*n* = 1), *Enterococcus faecium*/viridans group streptococci (*n* = 1), *Klebsiella pneumoniae*/*Enterobacter aerogenes* (*n* = 1), and methicillin-susceptible *S*. *aureus*/methicillin-resistant *Staphylococcus schleiferi* (*n* = 1).

There were some differences in the proportions of pathogens between community-acquired HVO and healthcare-associated HVO. MRSA was more frequent in healthcare-associated HVO than in community-acquired HVO (43.6% vs. 13.8%; *P* < 0.001), but MSSA was more frequent in community-acquired HVO than in healthcare-associated HVO (44.0% vs. 13.8%; *P* < 0.001). *Streptococcus* species were more frequent in community-acquired cases than in healthcare-associated cases (16.0% vs. 4.5%; *P* = 0.001). This trend was consistent across different *Streptococcus* species: viridans group streptococci (8.0% vs. 2.3%; *P* = 0.03), *Streptococcus agalactiae* (5.3% vs. 2.2%; *P* = 0.16) and *Streptococcus pneumoniae* (1.8% vs. 0%; *P* = 0.30).

### Antimicrobial susceptibility profiles of isolated organisms

The antimicrobial susceptibility testing results of isolated microorganisms are shown in Table 2. About 95% of MSSA isolates were susceptible to fluoroquinolones, compared to only half of the MRSA isolates. Of 69 *Enterobacteriaceae* isolates, 17 (24.6%) were resistant to ciprofloxacin, and 6 (8.7%) were extended-spectrum β-lactamase (ESBL) producers. All five *Pseudomonas aeruginosa* isolates were susceptible to ciprofloxacin. Of 42 *Streptococcus* species isolates, 40 (95.2%) were susceptible to amoxicillin-clavulanate and 38 (90.5%) were susceptible to levofloxacin. Of the 10 CoNS isolates, 5 (50%) were methicillin-resistant CoNS.

### Relevance of empirical antibiotic regimens for HVO

The relevance of empiric antibiotic regimens for HVO is shown in Table 3. Overall, 73.5% of isolated pathogens were susceptible to levofloxacin plus rifampicin, 71.2% to levofloxacin plus clindamycin, and 64.5% to amoxicillin-clavulanate plus ciprofloxacin. The susceptibility to these oral combinations was lower in cases of healthcare-associated HVO (52.6%, 49.6%, and 37.6%, respectively) than in cases of community-acquired HVO (85.8%, 84.0%, and 80.4%, respectively). Vancomycin combined with ciprofloxacin, ceftriaxone, ceftazidime, or cefepime was similarly appropriate (susceptibility rates 93.0%, 94.1%, 95.8%, and 95.8%, respectively). All antibiotic regimens gave similar results, irrespective patients’ age and blood culture positivity.

**Table 3:** Relevance of empiric antibiotic therapy based on the susceptibility results of organisms isolated from hematogenous vertebral osteomyelitis.

Organism	LEVO + RIF	LEVO + CLM	AMX-CL + CIP	CFZ^a^	VAN + CIP	VAN + CTR	VAN + CAZ	VAN + FEP
All cases (*n* = 358)	73.5	71.2	66.5	61.6	93.0	94.1	95.8	95.8
Microorganisms								
Methicillin-susceptible *S*. *aureus* (*n* = 120)	93.3	88.3	94.2	100	100	100	100	100
Methicillin-resistant *S*. *aureus* (*n* = 89)	50.6	41.6	0	0	100	100	100	100
Coagulase-negative staphylococci (*n* = 10)	70.0	70.0	50.0	50.0	100	100	100	100
*Streptococcus* species (*n* = 42)	90.5	97.6	100	100^b^	100	100	100	100
*Enterococcus* species (*n* = 10)	0	0	90.0	0	100	100	100	100
*Enterobacteriaceae* (*n* = 69)	75.4	75.4	78.3	82.6	75.4	91.3	91.3	91.3
*Pseudomonas aeruginosa* (*n* = 5)	100	100	0	60.0	100	0	100	100
Other (*n* = 13)	30.8	53.8	61.5	23.1	38.5	23.1	30.8	30.8
Onset of infection								
Community-acquired (*n* = 225)	85.8	84.0	80.4	78.3	94.2	96.9	97.3	97.3
Healthcare-associated (*n* = 133)	52.6	49.6	37.6	35.4	91.0	89.5	93.2	93.2
Age								
<65 years (*n* = 165)	77.6	72.7	69.1	67.8	93.3	93.9	95.8	95.8
≥65 years (*n* = 193)	69.9	69.9	60.6	56.5	92.7	94.3	95.9	95.9
Blood culture								
Non-bacteremic HVO (*n* = 93)^c^	77.4	79.6	66.7	61.4	91.7	86.0	92.5	92.5
Bacteremic HVO (*n* = 265)	72.1	68.3	63.8	61.6	92.8	97.0	97.0	97.0

Data are no. (%) of isolates, unless otherwise indicated.

Abbreviations: AMX-CL, amoxicillin-clavulanate; FEP, cefepime; CAZ, ceftazidime; CIP

ciprofloxacin; CFZ, cefazolin; CLM, clindamycin; CTR, ceftriaxone; HVO, hematogenous vertebral osteomyelitis; LEVO, levofloxacin; RIF, rifampin; VAN, vancomycin.

^a^ After excluding 25 streptococcal isolates without susceptibility data (21 viridans group streptococci and 4 *S*. *pneumoniae*), 333 isolates were included in analysis.

^b^ After excluding 25 streptococcal isolates without susceptibility data (21 viridans group streptococci and 4 *S*. *pneumoniae*), the remaining 17 *Streptococcus* isolates were susceptible to cefazolin.

^c^ Included patients with negative blood culture results (*n* = 74) and those from whom blood cultures were not obtained (*n* = 19).

## Discussion

We evaluated the susceptibility profiles of microorganisms causing HVO and assessed the appropriateness of several empiric antibiotic regimens for HVO. Our data showed that oral antibiotic combinations may be suboptimal for empiric treatment of HVO, especially in cases of healthcare-associated HVO. Vancomycin combined with fluoroquinolones or broad-spectrum cephalosporin may be appropriate for most cases of HVO.

In this study, the most frequently isolated organism was *S*. *aureus*, with 43% being MRSA. In recent years, the prevalence of MRSA among *S*. *aureus* vertebral osteomyelitis has been reported to be 40–57% [22,26,28,29]. The resistance rate of *Enterobacteriaceae* isolates to ciprofloxacin was 26% in this study, compared to 31% and 38% in two recent studies conducted in South Korea and France, respectively [30,31]. Vertebral osteomyelitis caused by antibiotic-resistant organisms may have a higher risk of treatment failure [28,32,33]. Indeed, patients with MRSA vertebral osteomyelitis reportedly have a 4–5-fold higher risk of recurrence than those with MSSA vertebral osteomyelitis [28,32]. In a study of patients with gram-negative bacterial HVO treated with 4–8 weeks of antibiotic treatment, 50% (4/8) of patients infected with fluoroquinolone-resistant strains experienced recurrence, but 16.7% (1/6) of patients infected with fluoroquinolone-resistant strains experienced recurrence [33]. Thus, the increasing incidence of antibiotic resistance in causative pathogens of vertebral osteomyelitis should be considered when selecting an empiric antibiotic regimen for HVO.

In seriously ill patients with suspected vertebral osteomyelitis, empiric antibiotic should be initiated in conjunction with an attempt at establishing a microbiologic diagnosis. We found that most of the isolated pathogens were susceptible to vancomycin plus ciprofloxacin or broad-spectrum cephalosporin. Although ESBL-producing *Enterobacteriaceae* accounted for 8.7% of the *Enterobacteriaceae* isolates, it accounted for only 1.7% of all causative organisms. The prevalence of anaerobes was 1.4% in this work, and up to 4% in two recent studies [2,34]. It may be underestimated because the successful isolation of anaerobic organisms largely depends on the quality of culture samples, mode of transport, and condition of culture. Despite this, antibiotic coverage of anaerobes (with carbapenems or metronidazole) may be not required initially, taking into account the rarity of these organisms and the risk of antibiotic overuse. It should be limited to patients who show no clinical improvement after empiric treatment and negative results of repeated biopsies. Patients with renal dysfunction or critical illness are at risk of developing vancomycin-induced nephrotoxicity, particularly when prolonged treatment is required. Alternative agents to vancomycin include teicoplanin, linezolid, daptomycin, and dalbavancin. Bone penetration was superior with teicoplanin but high dose therapy was required to treat staphylococcal osteomyelitis. Linezolid is recommended as an option for MRSA osteomyelitis, although the increasing risk of bone marrow suppression with prolonged courses of linezolid is a concern [8]. Recently, a retrospective analysis of 61 cases of MRSA vertebral osteomyelitis showed that patients treated with daptomycin had a higher cure rate than those treated with vancomycin (97% [30/31] vs 70% [21/30]; *P* = .006) [35].

In patients with no microbiological confirmation after routine diagnostic tests, several considerations should be made before starting empiric antibiotic therapy. First, *M*. *tuberculosis* and *Brucella* species should be excluded by appropriate diagnostic tests. Second, Concomitant infective endocarditis should be excluded in patients with underlying cardiac diseases. Bacteremic vertebral osteomyelitis caused by *Streptococcus* and *Enterococcus* species was frequently associated with infective endocarditis [36–39]. Third, there are some issues for use of fluoroquinolone-based regimens for treatment of staphylococcal vertebral osteomyelitis, although they have been frequently used not only in microbiologically proven cases [34,40], but also in cases of no microbiologic diagnosis [2,19,20]. Two previous studies showed that empiric treatment for vertebral osteomyelitis with levofloxacin plus rifampin had overall response rates of 78% (14/18) [2] and 84% (37/44) [19]. In another study, empiric treatment with ciprofloxacin plus amoxicillin-clavulanate had an overall response rate of 82% (18/22) [20]. Most of these studies were conducted in an epidemiologic setting featuring a low prevalence of MRSA [2,19,20]. In the current study, 64.5–73.5% of causative organisms were susceptible to fluoroquinolone conjunction with rifampin, clindamycin, or amoxicillin-clavulanate. The substantial rates of non-susceptibility to these regimens may be due to the high prevalence of MRSA (25%). Almost half of our MRSA isolates were resistant to fluoroquinolones, and the resistance rates to their companion drugs, such as rifampin and clindamycin, were high (22% and 58%, respectively). Fluoroquinolone plus rifampin are the most extensively studied combination for MSSA osteoarticular infections, but there is limited clinical experience of MRSA osteoarticular infections with such a combination [41,42]. Clinical guidelines recommend fluoroquinolone plus rifampin and clindamycin as acceptable alternatives for staphylococcal osteomyelitis, but they do not recommend fluoroquinolone monotherapy and oral β-lactams such amoxicillin-clavulanate and first-generation cephalosporin [8,43]. Fourth, in areas of high incidence of fluoroquinolone-resistant *Enterobacteriaceae*, fluoroquinolones should not be used especially if the patient has a previous history of healthcare-associated urinary tract infection or urinary tract infection due to fluoroquinolone-resistant *Enterobacteriaceae* [44,45]. Recently, Desoutter *et al*. reported the susceptibility patterns of microorganisms isolated from 101 cases of non-bacteremic vertebral osteomyelitis by percutaneous needle biopsy [30]. The isolated pathogens were susceptible to fluoroquinolone-based regimens (conjunction with rifampin, clindamycin, and amoxicillin-clavulanate) in 58–77% of cases [30]. In that study, in contrast to our study, MRSA was responsible for 5% of cases of vertebral osteomyelitis, but 39% of *Enterobacteriaceae* isolates was resistant to fluoroquinolone [30]. Finally, empiric antibiotic should be based on the host and the epidemiologic risk, as well as the local historical in vitro susceptibility data. To minimize the risk of microbiological failure due to resistant organisms, we reassessed the suitability of empiric regimens in community-acquired and healthcare-associated cases. These analyses indicated that overall resistance rate of isolated organisms to fluoroquinolone-based regimens was high (47–62%) in healthcare-associated cases and still substantial (14–20%) in community-acquired cases. Aguilar-Company reported that elderly patients with vertebral osteomyelitis had higher rates of healthcare-associated than younger patients [46], but we found no difference in overall susceptibility to empiric antibiotic regimens between both groups. Taken together, we suggest that fluoroquinolone-based oral combinations may be not appropriate due to frequent resistance to these agents, especially in cases of healthcare-associated HVO.

For culture-negative vertebral osteomyelitis, some authors suggest a first-generation cephalosporin for community-acquired cases and vancomycin-containing regimens for post-surgical cases, and have reported favorable outcomes with this approach [14,27]. A first-generation cephalosporin is an acceptable alternative in patients unable to tolerate anti-staphylococcal penicillin [47], but clinical experience with this agent for streptococcal and gram-negative bacterial osteomyelitis is limited. In addition, first-generation cephalosporin show varying activity against α-hemolytic streptococci, unlike β-hemolytic streptococci [48], and susceptibility testing of this agent against α-hemolytic streptococci is not recommended [24]. Even after excluding 25 cases of α-hemolytic streptococci without susceptibility data (viridans group streptococci [n = 21] and *S*. *pneumoniae* [n = 4]), cefazolin was appropriate in only 62% of cases. More data on the efficacy of first-generation cephalosporin is required before they can be widely used for culture-negative vertebral osteomyelitis.

Our study had several limitations. First, some cases lacked antibiotic susceptibility test results and so exclusion of these cases may have introduced bias. Second, we assessed the appropriateness of empiric antibiotic regimens for culture-negative HVO based on the data of organisms isolated from microbiologically proven cases. The bacterial etiology in culture-negative vertebral may differ according to diagnostic criteria and tests. Culture-negative vertebral osteomyelitis is less likely to be caused by *Staphylococcus* species, but it may be more likely to be caused by *Streptococcus* species [2,38]. Thus, our data may be more useful for selecting empiric antibiotics in seriously ill patients who require immediate antibiotic treatment than in those with no microbiological diagnosis. Despite this, we believe that our suggested regimens (vancomycin plus broad-spectrum cephalosporin or fluoroquinolone) may be also reasonable for the latter patients, when considering the risks of inappropriate antibiotic therapy and antibiotic overuse. Finally, this study included only patients with HVO, and so our findings should not be extrapolated to cases of post-surgical vertebral osteomyelitis, in which CoNS and antibiotic-resistant organisms may be more common.

In summary, in a setting in which MRSA is a frequent causative organism of HVO, cautious selection of empiric antibiotics is required. Oral antibiotic combinations may be suboptimal due to frequent resistance to these agents, especially in healthcare-associated cases. Vancomycin combined with fluoroquinolone or a broad-spectrum cephalosporin may be appropriate in most cases of HVO. It should be noted that our evaluation of the suitability of the empiric regimens for HVO was just based on overall susceptibility of isolated pathogens for microbiologically proven cases. Therefore, further studies are required to clarify the clinical impact of empiric regimens suggested here on patients’ outcomes.

## References

[1] Cottle L, Riordan T. Infectious spondylodiscitis. J Infect 2008; 56(6): 401–12. 10.1016/j.jinf.2008.02.005 18442854

[2] Lora-Tamayo J, Euba G, Narvaez JA, Murillo O, Verdaguer R, Sobrino B, et al Changing trends in the epidemiology of pyogenic vertebral osteomyelitis: the impact of cases with no microbiologic diagnosis. Semin Arthritis Rheum 2011; 41(2): 247–55. 10.1016/j.semarthrit.2011.04.002 21665246

[3] Akiyama T, Chikuda H, Yasunaga H, Horiguchi H, Fushimi K, Saita K. Incidence and risk factors for mortality of vertebral osteomyelitis: a retrospective analysis using the Japanese diagnosis procedure combination database. BMJ Open 2013; 3(3): e002412 10.1136/bmjopen-2012-002412 23533214 PMC3612742

[4] Kehrer M, Pedersen C, Jensen TG, Lassen AT. Increasing incidence of pyogenic spondylodiscitis: a 14-year population-based study. J Infect 2014; 68(4): 313–20. 10.1016/j.jinf.2013.11.011 24296494

[5] Pigrau C, Rodriguez-Pardo D, Fernandez-Hidalgo N, Moreto L, Pellise F, Larrosa MN, et al Health care associated hematogenous pyogenic vertebral osteomyelitis: a severe and potentially preventable infectious disease. Medicine (Baltimore) 2015; 94(3): e365. 25621677 10.1097/MD.0000000000000365 PMC4602631

[6] Renz N, Haupenthal J, Schuetz MA, Trampuz A. Hematogenous vertebral osteomyelitis associated with intravascular device-associated infections—A retrospective cohort study. Diagn Microbiol Infect Dis 2017; 88(1): 75–81. 10.1016/j.diagmicrobio.2017.01.020 28258789

[7] Gouliouris T, Aliyu SH, Brown NM. Spondylodiscitis: update on diagnosis and management. J Antimicrob Chemother 2010; 65 Suppl 3: iii11–24. 20876624 10.1093/jac/dkq303

[8] Berbari EF, Kanj SS, Kowalski TJ, Darouiche RO, Widmer AF, Schmitt SK, et al 2015 Infectious Diseases Society of America (IDSA) Clinical Practice Guidelines for the Diagnosis and Treatment of Native Vertebral Osteomyelitis in Adults. Clin Infect Dis 2015; 61(6): e26–46. 10.1093/cid/civ482 26229122

[9] Gras G, Buzele R, Parienti JJ, Debiais F, Dinh A, Dupon M, et al Microbiological diagnosis of vertebral osteomyelitis: relevance of second percutaneous biopsy following initial negative biopsy and limited yield of post-biopsy blood cultures. Eur J Clin Microbiol Infect Dis 2014; 33(3): 371–5. 10.1007/s10096-013-1965-y 24057139

[10] Marschall J, Bhavan KP, Olsen MA, Fraser VJ, Wright NM, Warren DK. The impact of prebiopsy antibiotics on pathogen recovery in hematogenous vertebral osteomyelitis. Clin Infect Dis 2011; 52(7): 867–72. 10.1093/cid/cir062 21427393 PMC3106232

[11] Kim CJ, Kang SJ, Yoon D, Lee MJ, Kim M, Song KH, et al Factors influencing culture positivity in pyogenic vertebral osteomyelitis patients with prior antibiotic exposure. Antimicrob Agents Chemother 2015; 59(4): 2470–3. 10.1128/AAC.04949-14 25666156 PMC4356833

[12] Chong BSW, Brereton CJ, Gordon A, Davis JS. Epidemiology, Microbiological Diagnosis, and Clinical Outcomes in Pyogenic Vertebral Osteomyelitis: A 10-year Retrospective Cohort Study. Open Forum Infect Dis 2018; 5(3): ofy037 10.1093/ofid/ofy037 29564362 PMC5846292

[13] Kim CJ, Song KH, Park WB, Kim ES, Park SW, Kim HB, et al Microbiologically and Clinically Diagnosed Vertebral Osteomyelitis: Impact of Prior Antibiotic Exposure. Antimicrobial Agents and Chemotherapy 2012; 56(4): 2122–4. 10.1128/AAC.05953-11 22232286 PMC3318334

[14] Kim J, Kim YS, Peck KR, Kim ES, Cho SY, Ha YE, et al Outcome of culture-negative pyogenic vertebral osteomyelitis: comparison with microbiologically confirmed pyogenic vertebral osteomyelitis. Semin Arthritis Rheum 2014; 44(2): 246–52. 10.1016/j.semarthrit.2014.04.008 24951277

[15] Chang WS, Ho MW, Lin PC, Ho CM, Chou CH, Lu MC, et al Clinical characteristics, treatments, and outcomes of hematogenous pyogenic vertebral osteomyelitis, 12-year experience from a tertiary hospital in central Taiwan. J Microbiol Immunol Infect 2017. 10.1016/j.jmii.2017.08.002 28847713

[16] de Lucas EM, Gonzalez Mandly A, Gutierrez A, Pellon R, Martin-Cuesta L, Izquierdo J, et al CT-guided fine-needle aspiration in vertebral osteomyelitis: true usefulness of a common practice. Clin Rheumatol 2009; 28(3): 315–20. 10.1007/s10067-008-1051-5 19043772

[17] Kim CJ, Kang SJ, Choe PG, Park WB, Jang HC, Jung SI, et al Which tissues are best for microbiological diagnosis in patients with pyogenic vertebral osteomyelitis undergoing needle biopsy? Clin Microbiol Infect 2015; 21(10): 931–5. 10.1016/j.cmi.2015.06.021 26119720

[18] Wu JS, Gorbachova T, Morrison WB, Haims AH. Imaging-guided bone biopsy for osteomyelitis: are there factors associated with positive or negative cultures? AJR Am J Roentgenol 2007; 188(6): 1529–34. 10.2214/AJR.06.1286 17515372

[19] Viale P, Furlanut M, Scudeller L, Pavan F, Negri C, Crapis M, et al Treatment of pyogenic (non-tuberculous) spondylodiscitis with tailored high-dose levofloxacin plus rifampicin. Int J Antimicrob Agents 2009; 33(4): 379–82. 10.1016/j.ijantimicag.2008.09.011 19097864

[20] Luzzati R, Giacomazzi D, Danzi MC, Tacconi L, Concia E, Vento S. Diagnosis, management and outcome of clinically- suspected spinal infection. J Infect 2009; 58(4): 259–65. 10.1016/j.jinf.2009.02.006 19268368

[21] von Elm E, Altman DG, Egger M, Pocock SJ, Gotzsche PC, Vandenbroucke JP, et al The Strengthening the Reporting of Observational Studies in Epidemiology (STROBE) statement: guidelines for reporting observational studies. Lancet 2007; 370(9596): 1453–7. 10.1016/S0140-6736(07)61602-X 18064739

[22] Livorsi DJ, Daver NG, Atmar RL, Shelburne SA, White AC Jr., Musher DM. Outcomes of treatment for hematogenous *Staphylococcus aureus* vertebral osteomyelitis in the MRSA ERA. J Infect 2008; 57(2): 128–31. 10.1016/j.jinf.2008.04.012 18562009

[23] Park KH, Cho OH, Lee JH, Park JS, Ryu KN, Park SY, et al Optimal Duration of Antibiotic Therapy in Patients With Hematogenous Vertebral Osteomyelitis at Low Risk and High Risk of Recurrence. Clin Infect Dis 2016; 62(10): 1262–9. 10.1093/cid/ciw098 26917813

[24] Clinical and Laboratory Standards Institute (2009) Performance Standards for Antimicrobial Susceptibility Testing; Nineteenth informational supplement; M100-S19 Wayne, PA, USA: Clinical and Laboratory Standards Institue.

[25] Clinical and Laboratory Standards Institute (2010) Performance Standards for Antimicrobial Susceptibility Testing; Twentieth informational supplement; M100-S20 Wayne, PA, USA: Clinical and Laboratory Standards Institue.

[26] Bhavan KP, Marschall J, Olsen MA, Fraser VJ, Wright NM, Warren DK. The epidemiology of hematogenous vertebral osteomyelitis: a cohort study in a tertiary care hospital. BMC Infect Dis 2010; 10: 158 10.1186/1471-2334-10-158 20529294 PMC2894835

[27] Yoon SH, Chung SK, Kim KJ, Kim HJ, Jin YJ, Kim HB. Pyogenic vertebral osteomyelitis: identification of microorganism and laboratory markers used to predict clinical outcome. Eur Spine J 2010; 19(4): 575–82. 10.1007/s00586-009-1216-1 19937064 PMC2899831

[28] Inoue S, Moriyama T, Horinouchi Y, Tachibana T, Okada F, Maruo K, et al Comparison of clinical features and outcomes of *staphylococcus aureus* vertebral osteomyelitis caused by methicillin-resistant and methicillin-sensitive strains. Springerplus 2013; 2(1): 283 10.1186/2193-1801-2-283 23853753 PMC3701790

[29] Weissman S, Parker RD, Siddiqui W, Dykema S, Horvath J. Vertebral osteomyelitis: retrospective review of 11 years of experience. Scand J Infect Dis 2014; 46(3): 193–9. 10.3109/00365548.2013.868600 24450841

[30] Desoutter S, Cottier JP, Ghout I, Issartel B, Dinh A, Martin A, et al Susceptibility Pattern of Microorganisms Isolated by Percutaneous Needle Biopsy in Nonbacteremic Pyogenic Vertebral Osteomyelitis. Antimicrob Agents Chemother 2015; 59(12): 7700–6. 10.1128/AAC.01516-15 26438497 PMC4649186

[31] Kang SJ, Jang HC, Jung SI, Choe PG, Park WB, Kim CJ, et al Clinical characteristics and risk factors of pyogenic spondylitis caused by gram-negative bacteria. PLoS One 2015; 10(5): e0127126 10.1371/journal.pone.0127126 25978839 PMC4433234

[32] Park KH, Chong YP, Kim SH, Lee SO, Choi SH, Lee MS, et al Clinical characteristics and therapeutic outcomes of hematogenous vertebral osteomyelitis caused by methicillin-resistant *Staphylococcus aureus*. J Infect 2013; 67(6): 556–64. 10.1016/j.jinf.2013.07.026 23916563

[33] Park KH, Cho OH, Jung M, Suk KS, Lee JH, Park JS, et al Clinical characteristics and outcomes of hematogenous vertebral osteomyelitis caused by gram-negative bacteria. J Infect 2014; 69(1): 42–50. 10.1016/j.jinf.2014.02.009 24561018

[34] Bernard L, Dinh A, Ghout I, Simo D, Zeller V, Issartel B, et al Antibiotic treatment for 6 weeks versus 12 weeks in patients with pyogenic vertebral osteomyelitis: an open-label, non-inferiority, randomised, controlled trial. Lancet 2015; 385(9971): 875–82. 10.1016/S0140-6736(14)61233-2 25468170

[35] Rangaraj G, KO. C, MS. G. Comparative analyhsis of daptomycin and vancomycin in the treatment of vertebral osteomeylitis. Inf Dis Clin Practice 2013; 22(4): 219–22.

[36] Pigrau C, Almirante B, Flores X, Falco V, Rodriguez D, Gasser I, et al Spontaneous pyogenic vertebral osteomyelitis and endocarditis: incidence, risk factors, and outcome. Am J Med 2005; 118(11): 1287. 10.1016/j.amjmed.2005.02.027 16271915

[37] Mulleman D, Philippe P, Senneville E, Costes C, Fages L, Deprez X, et al Streptococcal and enterococcal spondylodiscitis (vertebral osteomyelitis). High incidence of infective endocarditis in 50 cases. J Rheumatol 2006; 33(1): 91–7. 16395756

[38] Murillo O, Roset A, Sobrino B, Lora-Tamayo J, Verdaguer R, Jimenez-Mejias E, et al Streptococcal vertebral osteomyelitis: multiple faces of the same disease. Clin Microbiol Infect 2014; 20(1): O33–8. 10.1111/1469-0691.12302 23889700

[39] Koslow M, Kuperstein R, Eshed I, Perelman M, Maor E, Sidi Y. The unique clinical features and outcome of infectious endocarditis and vertebral osteomyelitis co-infection. Am J Med 2014; 127(7): 669 e9– e15. 10.1016/j.amjmed.2014.02.023 24608019

[40] Babouee Flury B, Elzi L, Kolbe M, Frei R, Weisser M, Scharen S, et al Is switching to an oral antibiotic regimen safe after 2 weeks of intravenous treatment for primary bacterial vertebral osteomyelitis? BMC Infect Dis 2014; 14: 226 10.1186/1471-2334-14-226 24767169 PMC4012835

[41] Coiffier G, Albert JD, Arvieux C, Guggenbuhl P. Optimizing combination rifampin therapy for staphylococcal osteoarticular infections. Joint Bone Spine 2013; 80(1): 11–7. 10.1016/j.jbspin.2012.09.008 23332140

[42] Kim BN, Kim ES, Oh MD. Oral antibiotic treatment of staphylococcal bone and joint infections in adults. J Antimicrob Chemother 2014; 69(2): 309–22. 10.1093/jac/dkt374 24072167

[43] Société de Pathologies Infectieuses de Langue Française (SPILF). Primary infectious spondylitis, and following intradiscal procedure, without prothesis. Recommendations. Med Mal Infect 2007; 37(9): 573–83. 18027462 10.1016/j.medmal.2007.03.007

[44] Bischoff S, Walter T, Gerigk M, Ebert M, Vogelmann R. Empiric antibiotic therapy in urinary tract infection in patients with risk factors for antibiotic resistance in a German emergency department. BMC Infect Dis 2018; 18(1): 56 10.1186/s12879-018-2960-9 29373965 PMC5787273

[45] Bosch-Nicolau P, Falco V, Vinado B, Andreu A, Len O, Almirante B, et al A Cohort Study of Risk Factors That Influence Empirical Treatment of Patients with Acute Pyelonephritis. Antimicrob Agents Chemother 2017; 61(12). 10.1128/AAC.01317-17 PMC5700303 28971876

[46] Aguilar-Company J, Pigrau C, Fernandez-Hidalgo N, Rodriguez-Pardo D, Falco V, Lung M, et al Native vertebral osteomyelitis in aged patients: distinctive features. An observational cohort study. Infection 2018. 10.1007/s15010-018-1177-6 30003490

[47] Lew DP, Waldvogel FA. Osteomyelitis. Lancet 2004; 364(9431): 369–79. 10.1016/S0140-6736(04)16727-5 15276398

[48] Fraimow HS. Systemic antimicrobial therapy in osteomyelitis. Semin Plast Surg 2009; 23(2): 90–9. 10.1055/s-0029-1214161 20567731 PMC2884905

